# Current use of statins reduces risk of HIV rebound on suppressive HAART

**DOI:** 10.1371/journal.pone.0172175

**Published:** 2017-03-01

**Authors:** Henning Drechsler, Colby Ayers, James Cutrell, Naim Maalouf, Pablo Tebas, Roger Bedimo

**Affiliations:** 1 Infectious Diseases Section, Medical Service, VA North Texas Health Care System, Dallas, Texas, United States of America; 2 Division of Infectious Diseases, Department of Medicine, University of Texas Southwestern School of Medicine, Dallas, Texas, United States of America; 3 Division of Biostatistics, Department of Clinical Sciences, University of Texas Southwestern School of Medicine, Dallas, Texas, United States of America; 4 Division of Infectious Diseases, Department of Medicine, University of Pennsylvania Perelman School of Medicine, Philadelphia, Pennsylvania, United States of America; University of Alabama at Birmingham, UNITED STATES

## Abstract

**Background:**

Despite compelling evidence for activity against HIV-1 *in vitro*, a virologic effect of statins has not been shown in clinical studies. Given their short plasma half-lives, such an effect may be transient and only apparent during ongoing exposure.

**Methods:**

We studied all HIV infected US-Veterans who started HAART 1995–2011, had a documented HIV viral load (VL) >1000 copies/mL, reached an undetectable VL on HAART, and had ≥1 follow-up VL within 13 months. We defined virologic failure (VF) as the first VL >1,000 copies/mL or the first of 2 consecutive VL >200 copies/mL. We built a time-updated drug exposure model for antiretrovirals (ARVs), statins, and other cardiovascular drugs (CVMs), investigating current use (yes/no), recent use (proportion of days used), and categorical use (ever/never). We used both multiply adjusted and inverse-probability-weighted (IPW) Cox models to explore the association between statin and CVM use and VF.

**Results:**

19,324 veterans met inclusion criteria. Median follow-up was 13 months (IQR: 5–32 months); 63% experienced VF after a median time of 9 months (IQR 4–21 months). Almost 1/3 patients ever used statins but exposure comprised only 41% of follow-up time covered after initial prescription. Unadjusted, current statin use was associated with a hazard ratio (HR) for VF of 0.60 (CI: 0.56–0.65). This remained statistically significant after multivariate adjustment (MVA) for demographics, HIV and HAART parameters [HR 0.81 (CI: 0.75–0.88), p<0.001] and IPW (truncation <1%/>99%) HR: 0.83 (CI: 0.75–0.92), p<0.001]. No independent association was observed for other CVMs. The association between categorical-statin use and VF after MVA was much weaker: HR 0.94 (CI: 0.88–1.00, p = 0.04).

**Conclusion:**

Current statin exposure was associated with reduced risk of VF in univariate, multivariate, and inverse-probability-weighted models. Our results highlight the importance of time-updated medication exposure models for observational studies.

## Introduction

Besides their cholesterol-lowering properties HMG-coenzyme A reductase inhibitors (statins) possess pleiotropic effects including improvement of endothelial dysfunction, increased nitric oxide production, antioxidant and anti-inflammatory properties, and stabilization of atherosclerotic plaques.[1] They also have *in vitro* antiviral effects [2, 3], most notably against hepatitis C virus (HCV) [4]. The anti HCV-effect has been clinically confirmed in a large retrospective study on US Veterans who had a 40–50% increased likelihood of achieving sustained virologic response with pegylated interferon and ribavirin treatment if they were concomitantly receiving statins. The effect was deemed independent of baseline hyperlipidemia itself, which also was found to be associated with an increased likelihood of virologic response [5].

Even before the recognition of the high prevalence of HIV- and highly active antiretroviral therapy (HAART)-related dyslipidemia [6], statins were known to interfere with HIV-1 replication *in vitro* [7]. Hypothesized molecular and cellular mechanisms for this include down-regulation of MHC-II on macrophages, interfering with viral entry via blocking ICAM-LFA1 interactions, disruption of CD4 CCR-5 expression, inhibition of HIV-1 integrase LEDGF/p75-HIV-1 interaction, or blocking of p21-mediated cell-cycle progression.[8–13]

Yet, clinical studies had never confirmed these *in vitro* data. In the absence of antiretroviral therapy (ART), statin use has not been associated with reduced HIV plasma viral load (VL) [14–16]. A European cohort analysis also showed no difference in virologic rebound rate for statin users after starting HAART, but did not account for current statin use or time to virologic rebound [17], and a smaller case-control study of 69 statin users showed a similar 1-year virologic suppression rate for statin users[18].

Given the relatively short plasma half-lives of statins, the absence of a clinically relevant virologic effect in these studies could be explained by not accounting for ongoing statin use. We hypothesized that a potential inhibitory effect on HIV replication would only be apparent in patients recently or currently exposed to statins. Given the low prevalence of hyperlipidemia and lipid-lowering therapy before virologic suppression [19] and the high potency of (current) HAART, an incremental effect of statins on initial virologic response may be difficult to detect. We instead investigated the effect of current and recent statin exposure on the risk of first virologic failure in patients who had already achieved virologic suppression on HAART.

## Materials and methods

### Data source

We used the VA Clinical Case Registry (CCR), which contains all non-narrative clinical data for HIV-infected patients receiving care in the Veterans Health Administration network [20]. It contains demographic data, laboratory values, vital signs, clinic utilization, detailed pharmacy data, procedure and diagnostic ICD-9 codes. The Institutional Review Board of the VA North Texas Health Care System approved this study and waived the requirement for written or verbal informed consent. We included all veterans who started HAART 1995–2011 if they achieved an undetectable VL (as defined below).

### Inclusion criteria and definitions

We studied all patients who had achieved virologic suppression on HAART from 1995–2011 meeting the following criteria: ≥ 1 detectable VL >1000 c/mL, followed by ≥ 1 undetectable VL, and ≥ 1 more subsequent VL measurement within 13 months after HAART initiation. Given that the threshold of lower limit of VL quantitation evolved of during the study period (from <500 in 1995 to <20 in 2011), patients were categorized as having undetectable VL according to the threshold in effect at the time of the measurement. For this purpose, ‘undetectable’ was defined as undetectable at any level <1000 copies/mL or <50 copies/mL. In a sensitivity analysis, we restricted inclusion to patients with ≥ 1 undetectable VL measurement after ≥ 6 months of viral suppression following HAART initiation. Virologic failure (VF) was defined analogous to current US guidelines [21] as the first VL measurement >1,000 c/mL or the first of two consecutive VL measurements >200 c/mL after initial virologic suppression.

### Follow-up time

Baseline was defined as the date of the first undetectable VL measurement after HAART initiation. Follow-up time ended at the day of VF, the last VL measurement before January 1 2012, or at death for the combined endpoint. We censored patients after 13 months without VL measurements to allow for one missed visit in patients with twice yearly monitoring. Premature or informative censoring was defined as censoring ≥13 months prior to January 1 2012.

### Outcomes measures / endpoints

1. Time to VF
2. Time to VF or death (within 13 months of last VL measurement).

### Stratification

We stratified all analyses by time during which first VL suppression was achieved, choosing three time-periods, which approximately amounted to tertiles and represented different HAART eras: 1) 1995–2000, 2) 2001–2005, and 3) 2005–2011.

### Covariates

We included the following baseline covariates: age, gender, race, history of drug abuse, time from HIV diagnosis to viral suppression, peak VL before baseline and duration of non-HAART ARV use prior to HAART initiation. We included the following time updated covariates: CD4 counts, ARV class experience, substance abuse, HCV status, and adherence to HAART components (see below). Most of these have been associated with VF [22]. Additionally, we explored whether plausible predictors of statin and CVM use were independently associated with VF including clinical history of cardiovascular disease, diabetes, smoking, LDL and non-HDL cholesterol, triglycerides, and body mass index (BMI).

### Medication exposure

Medication exposure and adherence for this study was derived from the VA pharmacy benefit management database, which is integrated into the CCR and contains detailed information about all outpatient medication prescriptions and refills and inpatient medication orders. Based on the assumption that patients were continuously exposed to filled outpatient medications until they ran out of drug supply, we tabulated uninterrupted exposure episodes on a day-to-day basis for antiretrovirals (ARVs), statins, non-statin lipid lowering agents (ALP), antihypertensives (AHT), and cardio-protective aspirin (ASA). We accounted for increased drug supply because of early refills, changes of therapy, and unused outpatient drugs during hospitalizations. During hospitalizations, medication exposure was complemented with inpatient prescription data. A detailed description of this process which is analogous to a recently proposed method for estimation of time-varying drug adherence [23] is contained in Text A in S1 File. At each day with a VL measurement, we determined three modes of medication exposure, which were carried forward to the next date with VL measurement:

1. Current use defined as current exposure/use within the previous 7 days
2. Proportion of days covered (PDC) measuring the extent of recent exposure, defined as *p*roportion of *d*ays *c*overed by drug exposure over the past 1 or 3 months.
3. Categorical use: all patients were classified as exposed after initial prescription.

The PDC model was calculated by subtracting cumulative medication exposure at days -30 or -91 from the cumulative medication exposure at the day of VL measurement and dividing the result by the corresponding time interval [24–26]. We used a 30-day PDC window for statins and CVMs and a 91-day PDC window for ARVs. PDC is an objective measure of medication adherence and a 90 day window for HAART adherence has been shown to accurately predict virologic failure [27].

We classified ARVs into nucleoside/nucleotide analogues (NRTIs) and anchor drugs (all other ARVs). We defined HAART as either: ≥1 anchor drug with ≥2 NRTIs, triple-class therapy, a protease inhibitor (PI) with ritonavir (bPI) plus a non-nucleoside reverse transcriptase inhibitor, or triple NRTI therapy if it contained: (tenofovir or abacavir) *and* zidovudine *and* (lamivudine or emtricitabine). For the purpose of comparative effectiveness, we grouped NRTI exposure into four complementary categories and anchor drug exposure into four mutually exclusive categories:

1. NRTI: a) Tenofovir; b) Lamivudine or Emtricitabine; c) other NRTIs; d) no NRTIs;
2. Anchor drug: a) Efavirenz, Ritonavir-boosted Darunavir, or Integrase Inhibitor (“modern anchor”); b) Ritonavir-boosted Protease Inhibitors (PI) other than darunavir; c) unboosted PI or Nevirapine or other; d) No anchor drug.

### Laboratory values and ICD-9 codes

We used VA National Laboratory Test codes and Logical Observation Identifier codes and custom text string searches to identify all relevant laboratory data. All laboratory values were handled as ‘last value carried forward’. We defined substance abuse at the time of virologic suppression as the presence of ≥ 2 indicative ICD9 codes. As statin initiation was likely guided by multiple and evolving criteria for hyperlipidemia, we created a unified parameter by calculating a low-density lipoprotein cholesterol (LDL) equivalent, which incorporated both LDL and non-high density (Non-HDL) cholesterol. This was defined as the maximum of LDL and (Non-HDL - 30mg/dL) for any cholesterol subclass measurement if the patient had been off lipid-lowering therapy >7 days.

### Statistical analysis

We fit Cox regression models with multivariate adjustment (MVA) for all medication exposure modes. We explored main effects of the stated covariates and retained all significant predictors (p<0.05) for the final models in which we also explored clinically plausible 2- and 3-way interactions between covariates (Tables B and C in S1 File). The proportionality assumption for all employed covariates was verified with Schoenfeld residuals.

We used generalized linear models with log-link function to determine the likelihood of current use of statins or CVMs or premature (informative) censoring. We first explored the main effects of all significant covariates from the MVA PDC model including time-updated age and retained only significant covariates for the final models in which we again included 2- and 3-way interactions including with time. The coefficients were then used to calculate propensity scores for current statin and CVM use and premature censoring. Based on current statin or CVM use or censoring we then calculated inverse proportional weights (IPW) for each time point with VL measurement which were stabilized by observed incidence and truncated at three different percentile levels: 5th, 1st and 0.1st percentile[28]. IPW was then used to weight Cox models for current statin and CVD use.

### Software

Data extraction, cleaning, and compilation and statistical procedures were carried out with SPSS (Version 23, IBM Corporation, Armonk, NY), Microsoft Excel for Mac and Windows 2011/2010, (Microsoft Corporation, Redmond, WA), and the survival package of R Version 3.31,(Foundation for Statistical Computing, Vienna, Austria) [29].

## Results

### Patient characteristics

Eighty percent of 36,360 veterans who started HAART from 1995–2011 achieved an undetectable VL measurement. Of these 29,112 patients, 19,324 met inclusion criteria. Their characteristics, stratified by HAART era during which viral suppression was achieved, are shown in Table 1.

**Table 1:** Baseline Characteristics *at the time of First VL Suppression*.

Median Values or percent *at day of initial VL suppression*		Overall	1995–2000	2001–2005	2006–2011
		n = 19,324	n = 6,678	n = 6,240	n = 6,406
VL assay detection limit < 50 copies/mL		58%	23%	61%	90%
Age		48 (42–54)	46 (40–52)	48 (42–54)	51 (44–57)
Female Gender		3%	2%	3%	3%
Race	African American	47%	39%	49%	54%
White		36%	34%	38%	36%
Unknown		15%	26%	11%	8%
HCV co-infection		26%	31%	27%	19%
Drug Abuse		33%	32%	34%	34%
>30 days of non HAART ARV experience		29%	52%	23%	10%
Months from HIV-Dx to viral suppression		31 (9–74)	33 (10–62)	34 (9–85)	28 (8–86)
VL before ART (log)		4.7 (4.0–5.2)	4.6 (4.0–5.2)	4.8 (4.1–5.3)	4.7 (4.0–5.1)
Peak VL (log)		5.0 (4.6–5.5)	5.0 (4.6–5.5)	5.1 (4.7–5.6)	5.0 (4.5–5.5)
CD4 count /mm^3^		333 (191–505)	321 (180–504)	311 (176–475)	367(221–531)
ARV class experience	2 classes	61%	72%	51%	59%
	3 classes	22%	20%	25%	22%
	4 classes	13%	7%	19%	13%
	5 classes	2%	0%	1%	4%
	6 classes	0%	0%	0%	1%
ART use last 3 months					
NRTIs	3TC/FTC	77%	76%	72%	84%
	TDF	31%	0%	23%	71%
	Other	61%	88%	74%	21%
Anchor	EFV / rDRV / INSTI	36%	14%	38%	57%
	boosted PI*	22%	6%	295	30%
	unboosted PI, NVP, or other	35%	75%	21%	7%
Native LDL equivalent** mg/dL		114 (89–143)	128 (98–160)	115 (89–145)	109 (87–134)
Body Mass Index		25.1 (22.4–28.3)	24.7 (22.2–27.6)	24.8 (22.2–28.0)	25.5 (22.6–28.9)
Statin use		7%	2%	7%	11%
Non Statin lipid-lowering agent use		4%	4%	6%	4%
Antihypertensive use		26%	20%	27%	32%
Cardiac Aspirin use		6%	5%	6%	7%

Medians (followed by IQR in parentheses) or percent (followed by %). EFV: efavirenz, rDRV: ritonavir boosted darunavir, INSTI: Integrase Inhibitor, NVP: nevirapine.

*other than rDRV

**Maximum of LDL cholesterol or Non-HDL cholesterol minus 30mg/dL when off lipid-lowering therapy.

The median observation time (time from suppression until last viral load measurement) was 5.9 years, inter-quartile range (IQR):2.6–9.8 years. In contrast, the median follow-up time (time until VF or censoring) was only 15 months (IQR: 6–40 months)). More than half of patients (55%, n = 10,534) experienced VF after a median time of 9 months (IQR 4–21 months) while 12% (n = 2,406) were prematurely censored. Twenty-two percent (n = 4,193) of study patients died. The majority of deaths (75%) occurred after virologic failure or censoring. Only 1,065 patients (25%) died during follow-up. Of note, 74% of the patients with VF subsequently achieved another episode of virologic suppression.

### Medication exposure

Almost three quarter of our patients (n = 14,016, 73%) used statins or CVMs during the study period follow up, most commonly AHT (n = 11,527, 60%), followed by statins (n = 6,502, 34%), ASA (n = 5,855, 30%), and ALP (n = 4,094, 21%). The most commonly used statin was pravastatin (40%), followed by simvastatin (23%), fluvastatin (13%), rosuvastatin (13%), atorvastatin (9%), and lovastatin (2%). Statins use was uncommon before reaching viral suppression; 91% of all statin users started statins while on suppressive HAART. Exposure to statins and CVMs was discontinuous. After initial prescription, the proportion of observation time subsequently covered by the respective medication class was only 60% for AHT, 41% for statins, 32% for ALP, and 27% for ASA. Specifically, exposure to statins was frequently interrupted, with almost half of all statin users having ≥1 exposure interruption of >14 days and one quarter having ≥5 such interruptions.

### Relation between statin and CVM use, HAART use, and risk of virologic failure

Table 2 contains a selection of time-dependent covariates at the last day with VL monitoring within each time period. Patients taking CVMs were older than those who were not. Patients on statins were more likely to be white (until 2005), while patients on AHT were more likely to be African American. In patients on CVMs, overall HAART adherence was higher throughout the study period, particularly for statins and ALP. In addition, fewer patients on lipid-lowering agents had a history of drug use.

**Table 2:** Cross-sectional Selection of Time-Updated Characteristics by Statin and CVM Use (all patients under observation).

At day of last VL measurement within time period (Median or %)		2000				2005				2011			
		Statin	ALP	AHT	none	Statin	ALP	AHT	none	Statin	ALP	AHT	none
		6%	4%	20%	70%	13%	5%	21%	61%	17%	2%	24%	57%
Median Age		52	50	52	47	55	51	54	49	58	56	56	51
(IQR)		(47–59)	(44–54)	(46–56)	(41–52)	(48–60)	(46–57)	(48–58)	(43–53)	(51–64)	(49–62)	(51–61)	(43–57)
Substance Use %		16%	19%	36%	32%	20%	29%	39%	33%	26%	18%	41%	32%
Race:	White	49%	63%	24%	37%	50%	61%	28%	39%	45%	55%	24%	38%
	African American	27%	24%	48%	41%	35%	29%	63%	51%	45%	30%	69%	52%
3 months HAART use rate: Mean [SD]		80 [30]	81 [30]	70 [33]	66 [35]	83 [27]	84 [28]	71 [33]	67 [36]	88 [23]	87 [22]	78 [30]	75 [33]
Proportion with >90%		67%	61%	45%	41%	67%	68%	49%	43%	72%	68%	54%	53%
On Tenofovir %		1%	0%	0%	0%	35%	41%	32%	35%	67%	69%	66%	68%
On EFV/rDRV/INSTI %		27%	25%	25%	21%	36%	27%	31%	29%	65%	69%	58%	54%
On boosted PI %		18%	15%	10%	10%	40%	53%	31%	31%	25%	23%	24%	24%
Median CD4		443	451	380	370	447	406	371	353	510	459	474	466
(IQR)		(315–632)	(302–639)	(225–577)	(211–575)	(290–654)	(291–621)	(228–538)	(211–522)	(345–677)	(310–582)	(300–661)	(293–642)

Medians (followed by IQR in parentheses), Mean [SD = standard deviation], or percent (followed by %). ALP: Non-Statin lipid-lowering agent use without concurrent statin use. AHT: Antihypertensive use without concurrent statin or ALP use, EFV: efavirenz, rDRV: ritonavir boosted darunavir, INSTI: Integrase Inhibitor, NVP: nevirapine.

In univariate analysis, current statin or CVM use was statistically associated with a decreased risk of VF. This was most pronounced for statins with a hazard ratio (HR) of 0.60, 95% Confidence Interval (CI): 0.56–0.65. After MVA, only statin use remained a significant negative predictor for VF: the adjusted HR was 0.83 (CI: 0.76–0.90) in the PDC model, and 0.81 (CI: 0.75–0.88) in the current use model. Using IPW, only statins but not CVMs were associated with lower VF risk: HR: 0.83 (CI: 0.75–0.92, 1% propensity score truncation [(Table 3)]). In contrast, current or recent use (PDC model) of cardio-protective aspirin was associated with an increased risk of VF after MVA but not IPW.

**Table 3:** Main Analysis. Hazard Ratio (95% CI) of first VF (n = 19,324 / 10, 534 failures).

Medication Exposure Mode	Bias Correction		Statins	Non-Statin Lipid—lowering Agents	Antihyper-tensives	Cardiac Aspirin
PDC (30/90d)*	None		0.57 (0.53–0.62)	0.76 (0.69–0.83)	0.79 (0.75–0.83)	0.83 (0.76–0.90)
			p<0.001	p<0.001	p<0.001	p<0.001
	Multivariate Adjustment		0.83 (0.76–0.90)	1.03 (0.94–1.14)	1.00 (0.95–1.05)	1.10 (1.01–1.21)
			p<0.001	p = 0.5	p = 0.85	p = 0.04
Current Use (within 7 d)	None		0.60 (0.56–0.65)	0.76 (0.70–0.83)	0.82 (0.79–0.86)	0.88 (0.81–0.95)
			p<0.001	p<0.001	p<0.001	p = 0.002
	Multivariate Adjustment		0.81 (0.75–0.88)		0.97 (0.89–1.06)	1.00 (0.96–1.05)	1.13 (1.04–1.23)	
			p<0.001		p = 0.53	p = 0.9	p = 0.004
	IPW	Truncation				
		<5% / >95%	0.76 (0.69–0.83)	0.97 (0.88–1.08)	0.98 (0.94–1.03)	1.01 (0.91–1.12)
			p<0.001	p = 0.64	p = 0.46	p = 0.86
		<1% / >99%	0.83 (0.75–0.92)	1.03 (0.92–1.15)	1.02 (0.97–1.07)	1.03 (0.92–1.15)
			p<0.001	p = 0.67	p = 0.57	p = 0.63
		<0.1%/>99.9%	0.90 (0.80–1.01)	1.04 (0.93–1.17)	1.03 (0.98–1.08)	1.04 (0.92–1.16)
			p = 0.08	p = 0.48	p = 0.29	p = 0.57

All hazard ratios (HR) are followed by 95% confidence intervals in parenthesis. PDC: percentage of days covered, IPW: Inverse Probability Weighting for treatment and censoring.

*PDC interval 30 days for statins/CVMs and 90 days for ARVs. In PDC mode, HR is for 100% use.

### Sensitivity analyses

A total of 14,841 patients were included in the sensitivity analysis restricted to patients with a virologic suppression period of at least 6 months. In this group, the median follow-up was 32 months (IQR 18–59 months), the rate of VF was 42% (n = 6,295), and the median time to VF was longer (23 months, IQR 15–39 months). The association between current statin use and reduced HR for VF was weaker but remained statistically significant after multivariate adjustment (HR 0.89, CI: 0.82–0.97) and IPW with 5% truncation (HR 0.85, CI: 0.78–0.94 [Table 4]). The current or recent use of cardio-protective aspirin was again independently associated (MVA) with an increased risk of VF.

**Table 4:** Sensitivity Analysis. Hazard Ratio (95% CI) for first VF after sustained viral suppression >6 months (n = 14,389 / 6,295 failures).

Medication Exposure Mode	Bias Correction		Statins	Non-Statin Lipid—lowering Agents	Antihyper-tensives	Cardiac Aspirin
PDC (30/90d)*	None		0.65 (0.60–0.70)	0.74 (0.67–0.82)	0.80 (0.75–0.84)	0.88 (0.80–0.97)
			p<0.001	p<0.001	p<0.001	p = 0.01
	Multivariate Adjustment		0.91 (0.83–0.99)	1.01 (0.90–1.12)	1.00 (0.94–1.06)	1.13 (1.02–1.25)
			p = 0.04	p = 0.9	p = 0.98	p = 0.02
Current Use (within 7 d)	None		0.67 (0.62–0.72)	0.75 (0.68–0.82)	0.83 (0.78–0.87)	0.91 (0.83–1.00)
			p<0.001	p<0.001	p<0.001	p = 0.04
	Multivariate Adjustment		0.89 (0.82–0.97)	0.95 (0.86–1.05)	1.00 (0.94–1.06)	1.12 (1.02–1.24)
			p = 0.007	p = 0.32	p = 0.97	p = 0.02
	IPW	Truncation				
		<5% / >95%	0.85 (0.78–0.94)	0.90 (0.79–1.02)	0.99 (0.93–1.05)	1.04 (0.92–1.17)
			p<0.001	p = 0.10	p = 0.69	p = 0.57
		<1% / >99%	0.93 (0.84–1.03)	0.95 (0.83–1.10)	1.02 (0.96–1.09)	1.06 (0.93–1.21)
			p = 0.18	p = 0.51	p = 0.46	p = 0.40
		<0.1%/>99.9%	1.00 (0.88–1.13)	0.97 (0.84–1.13)	1.04 (0.97–1.11)	1.07 (0.93–1.23)
			p = 0.97	p = 0.73	p = 0.24	p = 0.32

All hazard ratios (HR) are followed by 95% confidence intervals in parenthesis. PDC: percentage of days covered, IPW: Inverse Probability Weighting for treatment and censoring.

*PDC interval 30 days for statins/CVMs and 90 days for ARVs. In PDC mode, HR is for 100% use.

### Additional analyses

For the combined endpoint of VF or death during viral suppression the effect of statin use was similar to the primary analysis (Table D in S1 File). When examining statin use as a time-dependent categorical variable by treating all patients as permanently exposed after their first statin prescription, the negative association between statin use and VF weakened. The HR for the univariate exposure model was 0.78 (CI: 0.74–0.83), and 0.94 (CI: 0.88–1.00, p = 0.04) after MVA in the current use model. In the adjusted PDC model, the association was no longer significant (HR 0.96, CI: 0.90–1.02, p = 0.16).

We also analyzed the virologic effect of current use of individual statin compounds. Compared to the statin class, hazard ratios for virologic failure were comparable for pravastatin and lovastatin, superior for simvastatin and rosuvastatin, and inferior for fluvastatin and atorvastatin in univariate analysis. After multivariate adjustment for the same covariates as in the primary analysis, current use of three of the four most commonly used statins, pravastatin (40%), simvastatin (23%), and rosuvastatin (13%), remained statistically significant (S1 File Table E).

**Fig 1:**
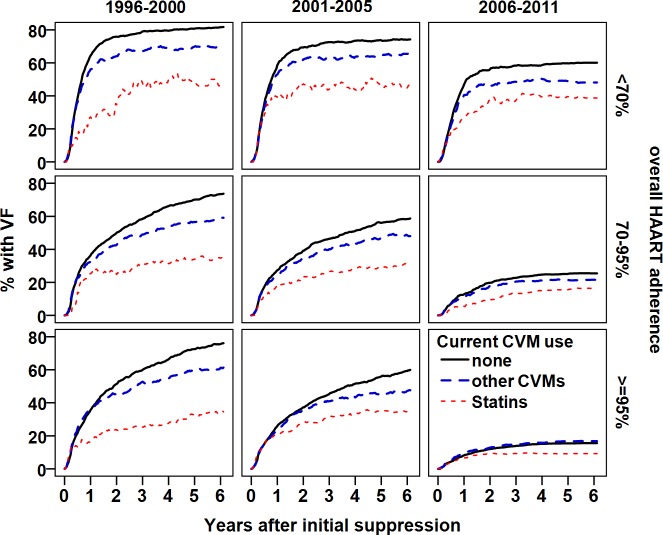
Proportion of patients with virological failure by overall HAART adherence since suppression (rows), and time period (columns). Solid line: not on statins or CVMs, broken line: other CVMs, dotted line: on statins. Statin use was associated with a decreased probability of virological failure.

**Fig 2:**
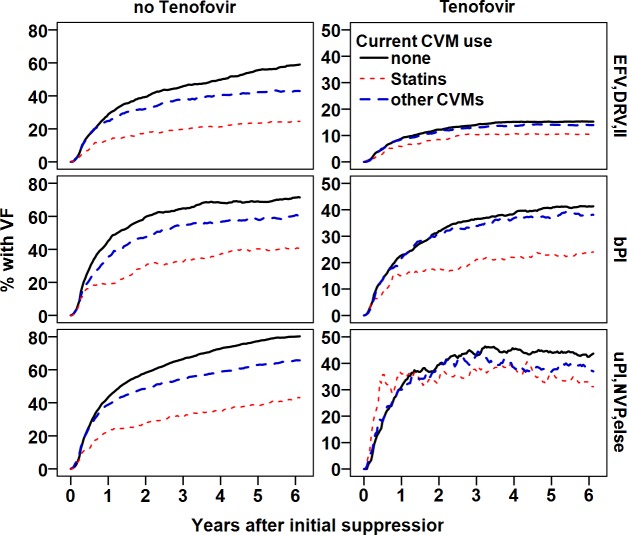
Proportion of patients with virological failure by predominant anchor drug (rows), and tenofovir exposure (>50%, columns) during the past 3 months. Solid line: not on statins or CVMs, broken line: on other CVMs, dotted line: on statins. Statin use was associated with a decreased probability of virological failure independently of the ART regimen used.

**Fig 3:**
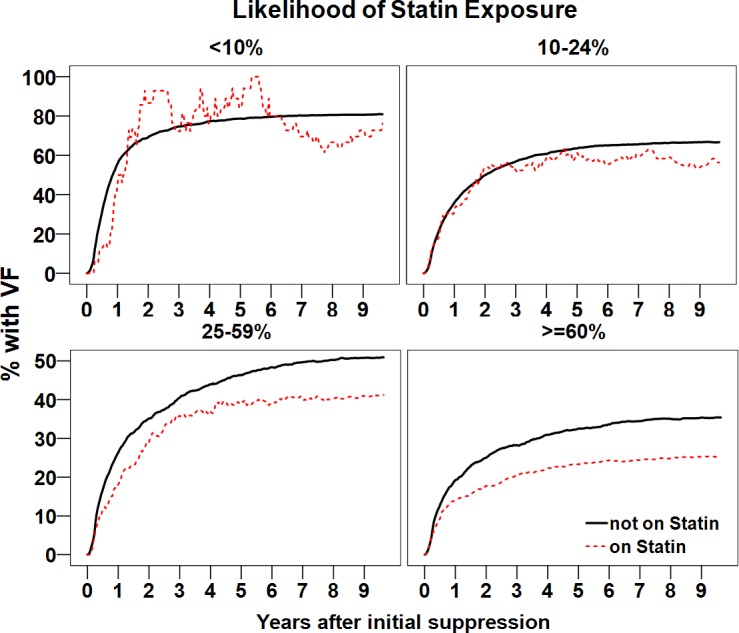
Proportion of patients experiencing virological failure by likelihood of statin exposure based on propensity score. Solid line: not on statins or CVMs, dotted line: on statins. Statin use was associated with a decreased probability of failure.

Figs 1–3 shows the proportion patients with initial VF over time, comparing statin users to users of other CVMs and patients without concurrent CVM or statin use. We stratified by time period of viral suppression and overall HAART adherence (Fig 1), current ARV use (Fig 2), and likelihood of statin use by propensity score (Fig 3).

## Discussion

We examined whether statin use during stable virologic suppression on HAART was independently associated with a lower risk of initial HIV rebound. We stratified all analyses by time period during which viral suppression was achieved. The three time periods chosen approximately amounted to tertiles and also represented different HAART eras. The first time period was characterized by the predominant use of unboosted PIs as anchor drugs, while the next time period from 2001–2005 was marked by the emergence of boosted PIs and tenofovir. The final period after 2005 saw the dominance of tenofovir as NRTI backbone, the abandonment of unboosted PIs as anchor drugs, and the emergence of fixed dose ARV combinations.

We found that current statin exposure was associated with a lower risk of VF in univariate and multivariate models and after inverse probability weighting. This was not seen with other CVMs, including ALPs, AHTs and ASA.

As illustrated in Figs 1 and 2 and Table E in S1 File, the negative effect of statins on VL rebound effect was present during the entire study period and among all strata of HAART adherence, and different types of HAART and statins taken; becoming less pronounced after 2005, in patients with optimal HAART adherence, and in those taking ‘contemporary’ HAART.

Because of the observational nature of our study and our strict definitions, virologic failure was common and often occurred early, leading to a short median follow-up time. This may have led to selection bias, as repeated confirmation of undetectable VL results may have often been a precondition for statin prescription. However, a sensitivity analysis restricted to patients with more than six months of virologic suppression also showed statistical significance, albeit with reduced effect size (HR 0.89 vs. 0.81). This may be related to the observation that the risk of virologic rebound declines with longer time of viral suppression, independent of HAART-adherence [30]. Patients on statins were older and more prone to pre-existing cardiovascular disease. Therefore, an increased death rate could have been a competing risk for VF. Yet, the analysis of the combined endpoint of VF or death yielded identical results than for VF alone.

When statin exposure was used as a time-dependent categorical variable (considering all patients permanently exposed after initial statin prescription), the association between statin use and viral rebound was much weaker (current use model) or no longer significant (PDC model). The likely explanation for this is that after initial prescription, actual statin exposure covered only 41% of observation time. A modest and transient effect would thus likely weaken after cessation of the drug. This may also be the explanation why our results of current and recent statin use are different from previous observational studies when statin use was analyzed as a categorical variable[31].

HAART adherence is the most important factor determining the risk of VF, and pharmacy refill data have emerged as a preferred mode of ARV adherence measurement [27, 32]. Our results highlight the importance of time-updated medication exposure models for observational studies.

The mechanism(s) of the protective effect of statins on the chance HIV rebound are unclear. While anti-inflammatory properties are arguably the most often cited pleiotropic statin effect and could play an important role in explaining our findings, we also observed that the use of cardio-protective aspirin was independently associated with a moderately increased risk of VF. An ongoing large controlled prospective trial with pitavastatin (REPRIEVE) [33] may soon shed more light on the existence and mechanisms of antiviral or advantageous immuno-modulatory effects of statins during HAART. If this can be confirmed, it may also be relevant for HIV eradication research as latency-reversing agents are thought to be insufficient without sufficient stimulation of cellular immune responses [34].

While a 17–19% relative risk reduction of VF would only translate into 3.5–4% absolute risk reduction in the context of a contemporary antiretroviral regimen with a virologic suppression rate of 80% of, our findings could have clinical importance in settings with sub-optimal adherence or limited options for a secondary ART regimen.

The strengths of our analysis include the rigorous and sophisticated modeling of medication exposure, owing to the very granular pharmacy utilization data of a large, well-characterized observational cohort. This allowed us to model daily use of concurrent ARVs, statins, and other CVMs, including other lipid-lowering agents and to analyze their effects concomitantly. We explored different modes of medication exposure, applied several forms of bias correction (MVA and IPW), and tested the robustness of our findings with a sensitivity analysis and an alternative outcome.

Cyclooxygenase-2 inhibitors have previously been reported to decrease markers of immune activation and cell exhaustion and increase immunologic responses to vaccination in HIV-infected adults in vivo[35], and to enhance virologic activity of zidovudine *in vitro* [36]. In our study, an unexpected finding was that current and recent exposure to cardio-protective aspirin was independently associated with a 10% increased chance of VF after MVA. This finding warrants further investigation and we speculate it could be due aspirin initiation at a time of an acute event (infection, cardiovascular) that may be linked to decreased adherence.

The limitations of our study include its retrospective and observational nature, the likely presence of unknown and/or unmeasured confounders, which we tried to address using IPW, and the lack of representation of women. In addition, our medication exposure model including the grouping of ARVs has not been validated and we did not have information on antiretroviral resistance for our models. The VA CCR is not an actively managed cohort and ARV exposure histories from outside the VA system are not captured within its pharmacy data, but the proportion of veterans obtaining antiretrovirals outside the system is very low. The precise time to virologic failure in clinical settings cannot be determined. Low medication adherence will affect the frequency of VL monitoring and patients with low HAART adherence may have been monitored more frequently and were thus likely to experience VF earlier in our model. While this would lead to an overestimation of the contribution of low medication adherence to VF, it may have been counterbalanced by delayed VL monitoring in other patients with poor adherence.

In summary, we show that statin use is independently associated with a lower risk of virologic rebound on HAART. To our knowledge, this is the first demonstration of such an adjuvant anti-HIV effect for a non-antiretroviral class of medications and may be another reason beyond cardiovascular benefits to use statins in HIV-infected individuals.

## Supporting information

S1 Data Contains the patient data of the file that was used for all analyses in tab delimited form, including a variable dictionary. (ZIP)

S1 File Text A: Describes the Medication Exposure Models in detail. Table A Legend: HR: none. Table B Legend: HR: Hazard Ratio (95% confidence interval in parenthesis). PDC: percentage of days covered: last 30 days for statins and CVMs, last, 90 days for ARVs, VF: Virologic Failure, Boosted PI: Ritonavir boosted Protease Inhibitor, 3TC: Lamivudine, FTC: Emtricitabine. Modern Anchor: Efavirenz, boosted Darunavir, or Integrase Inhibitor. Gray Shade: Baseline covariates (non-time dependent). Native LDL equivalent: LDL or Non-HDL cholesterol -30mg/dL, whichever was higher off lipid-lowering agents. Treatment Interruption: Time period without any ARVs. P-values: * 0.01–0.05, ** 0.001–0.01, *** <0.001. Table C Legend: HR: Hazard Ratio (95% confidence interval in parenthesis). PDC: percentage of days covered: last 30 days for statins and CVMs, last, 90 days for ARVs, VF: Virologic Failure, Boosted PI: Ritonavir boosted Protease Inhibitor, 3TC: Lamivudine, FTC: Emtricitabine. Modern Anchor: Efavirenz, boosted Darunavir, or Integrase Inhibitor. Gray Shade: Baseline covariates (non-time dependent). Native LDL equivalent: LDL or Non-HDL cholesterol -30mg/dL, whichever was higher off lipid-lowering agents. Treatment Interruption: Time period without any ARVs. P-values: * 0.01–0.05, ** 0.001–0.01, *** <0.001. Table D Legend: All hazard ratios (HR) are followed by 95% confidence intervals in parenthesis.PDC: percentage of days covered, IPW: Inverse Probability Weighting for treatment and censoring. *PDC interval 30 days for statins/CVMs and 90 days for ARVs. In PDC mode HR is for 100% use. Table E Legend: All hazard ratios (HR) are followed by 95% confidence intervals in parenthesis.
(DOCX)
