# Supplementary material for: Current use of statins reduces risk of HIV rebound on suppressive HAART
Source: PLoS One. 2017 Mar 1;12(3):e0172175. doi: 10.1371/journal.pone.0172175 (PMC5331966; doi:10.1371/journal.pone.0172175)
Supplement: S1 File — Text A: Describes the Medication Exposure Models in detail. Table A Legend: HR: none. Table B Legend: HR: Hazard Ratio (95% confidence interval in parenthesis). PDC: percentage of days covered: last 30 days for statins and CVMs, last, 90 days for ARVs, VF: Virologic Failure, Boosted PI: Ritonavir boosted Protease Inhibitor, 3TC: Lamivudine, FTC: Emtricitabine. Modern Anchor: Efavirenz, boosted Darunavir, or Integrase Inhibitor. Gray Shade: Baseline covariates (non-time dependent). Native LDL equivalent: LDL or Non-HDL cholesterol -30mg/dL, whichever was higher off lipid-lowering agents. Treatment Interruption: Time period without any ARVs. P-values: * 0.01–0.05, ** 0.001–0.01, *** <0.001. Table C Legend: HR: Hazard Ratio (95% confidence interval in parenthesis). PDC: percentage of days covered: last 30 days for statins and CVMs, last, 90 days for ARVs, VF: Virologic Failure, Boosted PI: Ritonavir boosted Protease Inhibitor, 3TC: Lamivudine, FTC: Emtricitabine. Modern Anchor: Efavirenz, boosted Darunavir, or Integrase Inhibitor. Gray Shade: Baseline covariates (non-time dependent). Native LDL equivalent: LDL or Non-HDL cholesterol -30mg/dL, whichever was higher off lipid-lowering agents. Treatment Interruption: Time period without any ARVs. P-values: * 0.01–0.05, ** 0.001–0.01, *** <0.001. Table D Legend: All hazard ratios (HR) are followed by 95% confidence intervals in parenthesis.PDC: percentage of days covered, IPW: Inverse Probability Weighting for treatment and censoring. *PDC interval 30 days for statins/CVMs and 90 days for ARVs. In PDC mode HR is for 100% use. Table E Legend: All hazard ratios (HR) are followed by 95% confidence intervals in parenthesis. (DOCX) [file pone.0172175.s002.docx]

Current Use of Statins Reduces Risk of HIV Rebound on Suppressive HAART Supplement

# S1 File

## Text A) Medication Exposure

Using outpatient fill and refill data (number of days supplied) as well as inpatient prescriptions, we tabulated exposure episodes for antiretrovirals (ARVs), statins, and different classes of cardio­vascular medications (CVMs). We calculated day-to-day exposure episodes for individual medications or fixed dose combination (FDC) of the following classes of medications:

1. ARVs: 34 medications (7 FDCs): 28 compounds
2. Statins: 7 medications (1 FDC): 6 compounds
3. Antihypertensives (AHT): 64 medications (17 FDCs): 47 compounds
4. Non-Statin lipid-lowering agents (ALP): 18 medications (1 FDC):10 compounds
5. Cardiac Aspirin (ASA): 1 compound. To exclude aspirin use for pain or fever we excluded all doses >325mg, and any prescriptions containing terms like ‘fever‘, ‘pain’, ‘ache’, ‘as needed’, or “PRN’ in the signature.

We then determined whether the prescription of a new medication was likely to result in the discontinuation of another medication of the same class. For example, the prescription of an FDC was deemed to terminate the previous exposure to any of its component previously taken as mono-substance or another FDC. Another example is the prescription of a different statin. For this purpose, we divided classes 1) to 4) into subclasses and modeled concurrent fills within the same subclass as consecutive exposures. Supplemental Table 1 lists the subclasses used to differentiate between consecutive and concurrent exposures and supplemental Suppl. Figure 1 illustrates the principles of the translation process.

We defined the start date of any exposure as: a) day of fill for outpatient fill at a VA facility (window fill) or inpatient prescription, b) 3^rd^ day after mail order fill. We defined the stop date (first day of non-exposure) as either: a) day after hospitalization without corresponding inpatient prescription, b) day of outpatient fill of a competing drug within the same subclass, or c) the day after the accumulated drug supply was exhausted, taking into account the accumulated drug supply resulting from early refills and hospitalizations. During hospitalizations, only inpatient prescriptions were counted towards exposure. For statins and ARVs, we allowed for drug recycling: We defined ‘spare supply’ resulting from early discontinuation after prescription of another medication of the same subclass and counted it towards overall drug supply for the next exposure episode if the original medication was refilled or re-prescribed within one year. Finally, to determine overall class exposure, we translated exposure to fixed dose combinations into individual compounds and then merged exposures back into subclasses (AHT or ALP only).

## S1 File Table A:

## Drug Subclasses modeled as consecutive exposures

| Antiretrovirals |
| --- |
| 1. Zidovudine / Stavudine |
| 1. Emtricitabine / Lamivudine |
| 1. NNRTIs |
| 1. Fosamprenavir / Amprenavir |
| 1. All Fixed Dose Combinations (FDC): compound or class overlap between FDCs and/or contained monosubstance |
| **Statins:** All compounds |
| Antihypertensives |
| 1. Beta Blockers |
| 1. Angiotensin Receptor Blockers /  Angiotensin Converting Enzyme Inhibitors |
| 1. Calcium Channel Blockers |
| 1. Thiazide Diuretics |
| 1. Miscellaneous (5a-f each treated as subclass) |
| 5a) Clonidine |
| 5b) Methyldopa |
| 5c) Isosorbide Mono or Dinitrate |
| 5d) Spironolactone |
| 5e) Reserpine |
| 5f) Hydralazine |
| 1. FDC: Any compound or subclass overlap between FDCs and corresponding monosubstance was modeled as consecutive exposure |
| Non-Statin Lipid—lowering Therapy |
| 1. All Fibrates |
| 1. All Bile Acid Sequesters |
| 1. All Fish Oils |
| 1. All Lipid Absorption Inhibitors |
| 1. All Niacin Preparations |

## S1 File Table B: Cox Model based on Recent Medication Exposure (PDC)

| Covariate/Factor | HR of VF (95% CI) | p-value |  |
| --- | --- | --- | --- |
| Statin PDC (30 days) | 0.83 (0.76-0.90) | <0.001 | *** |
| ALP PDC (30 days) | 1.03 (0.94-1.14) | 0.5 |  |
| AHT PDC (30 days) | 1.00 (0.95-1.05) | 0.85 |  |
| ASA PDC (30 days) | 1.10 (1.01-1.21) | 0.04 | * |
| Peak VL (per log >3) | 1.45 (1.41-1.50) | <0.001 | *** |
| AGE at VL suppression | 0.91 (0.89-0.93) | <0.001 | *** |
| Drug Use at Baseline | 1.06 (1.01-1.10) | 0.008 | ** |
| Cumulative non-HAART ARV experience before HAART (per y) | 1.34 (1.24-1.44) | <0.001 | *** |
| Time from HIV diagnosis to suppression (per y) | 1.02 (1.02-1.03) | <0.001 | *** |
| Cum non-HAART ARV experience * Time from HIV diagnosis | 0.97 (0.94-0.99) | 0.006 | ** |
| ARV class experience (per class) | 1.03 (1.00-1.07) | 0.09 | . |
| ARV class experience * Cumulative non-HAART ARV experience | 0.98 (0.98-0.99) | <0.001 | *** |
| LDL equivalent (per 10 mg/dL) | 1.03 (1.01-1.05) | 0.002 | ** |
| BMI (minus 10) | 1.02 (1.01-1.04) | 0.001 | ** |
| LDL equivalent * BMI (minus 10) | 0.99 (0.99-1.00) | <0.001 | *** |
| Last CD4 count (per 100/mm^3^) | 0.89 (0.88-0.90) | <0.001 | *** |
| Treatment Interruption PDC (90 days) | 1.16 (0.98-1.37) | 0.08 | . |
| Modern Anchor PDC (90 days) | 0.25 (0.20-0.31) | <0.001 | *** |
| Boosted PI Anchor PDC (90 days) | 0.64 (0.57-0.71) | <0.001 | *** |
| Other Anchor PDC (90 days) | 0.91 (0.78-1.07) | 0.27 |  |
| Tenofovir PDC (90 days) | 0.69 (0.51-0.93) | 0.02 | * |
| 3TC/FTC PDC (90 days) | 0.77 (0.62-0.95) | 0.02 | * |
| Other NRTI PDC (90 days) | 0.44 (0.36-0.54) | <0.001 | *** |
| Tenofovir PDC * 3TC/FTC PDC | 0.57 (0.40-0.83) | 0.003 | ** |
| Tenofovir PDC * Other NRTI PDC | 0.88 (0.60-1.27) | 0.49 |  |
| 3TC/FTC PDC * Other NRTI PDC | 0.68 (0.54-0.86) | 0.002 | ** |
| ARV class experience * Modern Anchor PDC | 1.16 (1.09-1.23) | <0.001 | *** |
| ARV class experience * Other Anchor PDC | 1.19 (1.11-1.28) | <0.001 | *** |
| Treatment Interruption PDC * Other Anchor PDC | 2.66 (1.78-3.97) | <0.001 | *** |
| Treatment Interruption PDC * Tenofovir PDC | 25.9 (12.4-53.9) | <0.001 | *** |
| Treatment Interruption PDC * 3TC/FTC PDC | 1.48 (0.94-2.33) | 0.09 | . |
| Treatment Interruption PDC * Other NRTI PDC | 2.08 (1.46-2.96) | <0.001 | *** |
| Tenofovir PDC * 3TC/FTC PDC * Other NRTI PDC | 3.22 (2.02-5.13) | <0.001 | *** |
| Tenofovir PDC * 3TC/FTC PDC * Treatment Interruption PDC | 0.02 (0.01-0.08) | <0.001 | *** |

***S1 File Table B Legend:*** *HR: Hazard Ratio (95% confidence interval in parenthesis). PDC: percentage of days covered: last 30 days for statins and CVMs, last, 90 days for ARVs, VF: Virologic Failure, Boosted PI: Ritonavir boosted Protease Inhibitor, 3TC: Lamivudine, FTC: Emtricitabine. Modern Anchor: Efavirenz, boosted Darunavir, or Integrase Inhibitor. Gray Shade: Baseline covariates (non-time dependent). Native LDL equivalent: LDL or Non-HDL cholesterol -30mg/dL, whichever was higher off lipid-lowering agents. Treatment Interruption: Time period without any ARVs. P-values: * 0.01-0.05, ** 0.001-0.01, *** <0.001*.

## S1 File Table C: Cox Model based Current Medication Exposure (last 7 d)

| Covariate | HR of VF (95% CI) | p-value |  |
| --- | --- | --- | --- |
| Statin | 0.81 (0.75-0.88) | <0.001 | *** |
| ALP | 0.97 (0.89-1.06) | 0.53 |  |
| AHT | 1.00 (0.96-1.05) | 0.90 |  |
| ASA | 1.13 (1.04-1.23) | 0.004 | ** |
| Peak VL (per log >3) | 1.44 (1.40-1.49) | <0.001 | *** |
| Age at VL suppression | 0.89 (0.87-0.91) | <0.001 | *** |
| Drug Use at Baseline | 1.11 (1.06-1.15) | <0.001 | *** |
| Time from HIV diagnosis to suppression (per y) | 1.03 (1.02-1.03) | <0.001 | *** |
| Cumulative non-HAART ARV experience before HAART (per y) | 1.32 (1.23-1.42) | <0.001 | *** |
| Time to suppression * Cumulative non-HAART ARV experience | 0.98 (0.98-0.99) | <0.001 | *** |
| Last CD4 count (per 100/mm^3^) | 0.89 (0.88-0.89) | <0.001 | *** |
| ARV class experience (per class) | 0.75 (0.47-1.20) | 0.23 |  |
| boosted PI Anchor (Ref.=Modern Anchor) | 0.10 (0.01-1.28) | 0.08 | . |
| Other Anchor (Ref.=Modern Anchor) | 0.28 (0.02-3.73) | 0.34 |  |
| No Anchor (Ref.=Modern Anchor) | 0.72 (0.12-4.34) | 0.72 |  |
| TDF+3TC/FTC (Ref.=Triple NRTIs) | 0.10 (0.02-0.45) | 0.002 | ** |
| Other NRTI+TDF (Ref.=Triple NRTIs) | 0.20 (0.03-1.53) | 0.12 |  |
| Other NRTI+3TC/FTC (Ref.=Triple NRTIs) | 0.09 (0.02-0.39) | 0.001 | ** |
| TDF only (Ref.=Triple NRTIs) | 0.11 (0.03-0.47) | 0.003 | ** |
| 3TC/FTC only (Ref.=Triple NRTIs) | 1.09 (0.08-15.3) | 0.95 |  |
| Other NRTI only (Ref.=Triple NRTIs) | 0.34 (0.07-1.52) | 0.16 |  |
| No NRTI (Ref.=Triple NRTIs) | 0.24 (0.06-1.00) | 0.05 | * |
| LDL equivalent (per 10 mg/dL) | 1.03 (1.01-1.05) | 0.001 | ** |
| BMI (minus 10) | 1.02 (1.01-1.04) | 0.002 | ** |
| ARV class experience * boosted PI Anchor | 2.02 (0.96-4.24) | 0.06 | . |
| ARV class experience * Other Anchor | 1.70 (0.74-3.87) | 0.21 |  |
| ARV class experience * No Anchor | 1.30 (0.69-2.47) | 0.41 |  |
| ARV class experience * TDF+3TC/FTC | 1.55 (0.95-2.53) | 0.08 | . |
| ARV class experience * Other NRTI+TDF | 1.46 (0.77-2.75) | 0.25 |  |
| ARV class experience * Other NRTI+3TC/FTC | 1.88 (1.15-3.06) | 0.01 | * |
| ARV class experience * TDF only | 1.69 (1.06-2.72) | 0.03 | * |
| ARV class experience * 3TC/FTC only | 0.93 (0.40-2.16) | 0.87 |  |
| ARV class experience * Other NRTI only | 1.41 (0.85-2.34) | 0.18 |  |
| ARV class experience * No NRTI | 1.52 (0.95-2.43) | 0.08 | . |
| boosted PI Anchor * TDF+3TC/FTC | 10 (0.7-146) | 0.09 | . |
| Other Anchor * TDF+3TC/FTC | 3.1 (0.2-53) | 0.43 |  |
| No Anchor * TDF+3TC/FTC | 4.6 (0.3-70) | 0.27 |  |
| boosted PI Anchor * Other NRTI+TDF | 9.8 (0.4-256) | 0.17 |  |
| Other Anchor * Other NRTI+TDF | 4.4 (0.07-268) | 0.48 |  |
| No Anchor * Other NRTI+TDF | 0.3 (0.01-18.8) | 0.58 |  |
| boosted PI Anchor * Other NRTI+3TC/FTC | 18.5 (1.3-265) | 0.03 | * |
| Other Anchor * Other NRTI+3TC/FTC | 7.5 (0.5-105) | 0.13 |  |
| No Anchor * Other NRTI+3TC/FTC | 5.9 (0.9-39.7) | 0.07 | . |
| boosted PI Anchor * TDF only | 30.4 (2.3-404) | 0.01 | ** |
| Other Anchor * TDF only | 9.0 (0.6-127) | 0.10 |  |
| No Anchor * TDF only | 5.7 (0.9-37.2) | 0.07 | . |
| boosted PI Anchor * 3TC/FTC only | 23.8 (0.5-1054) | 0.10 |  |
| Other Anchor * 3TC/FTC only | 1.3 (0.03-52) | 0.89 |  |
| No Anchor * 3TC/FTC only | 1.8 (0.06-60) | 0.73 |  |
| boosted PI Anchor * Other NRTI only | 11.1 (0.7-175) | 0.09 | . |
| Other Anchor * Other NRTI only | 4.1 (0.3-59) | 0.30 |  |
| No Anchor * Other NRTI only | 3.4 (0.5-24) | 0.22 |  |
| boosted PI Anchor * No NRTI | 11.4 (0.9-151) | 0.06 | . |
| Other Anchor * No NRTI | 4.6 (0.3-61) | 0.25 |  |
| No Anchor * No NRTI | 6 (0.99-36.5) | 0.05 | . |
| LDL equivalent * BMI minus 10 | 0.99 (0.99-1) | <0.001 | *** |
| ARV class experience * Non-HAART ARV experience | 0.97 (0.95-0.99) | 0.01 | * |
| ARV class experience * boosted PI Anchor * TDF+3TC/FTC | 0.57 (0.26-1.24) | 0.16 |  |
| ARV class experience * Other Anchor * TDF+3TC/FTC | 0.77 (0.31-1.91) | 0.58 |  |
| ARV class experience * No Anchor * TDF+3TC/FTC | 0.74 (0.31-1.77) | 0.50 |  |
| ARV class experience * boosted PI Anchor * Other NRTI+TDF | 0.59 (0.23-1.52) | 0.27 |  |
| ARV class experience * Other Anchor * Other NRTI+TDF | 0.66 (0.18-2.36) | 0.52 |  |
| ARV class experience * No Anchor * Other NRTI+TDF | 1.86 (0.54-6.39) | 0.32 |  |
| ARV class experience * boosted PI Anchor * Other NRTI+3TC/FTC | 0.46 (0.21-1.00) | 0.05 | * |
| ARV class experience * Other Anchor * Other NRTI+3TC/FTC | 0.56 (0.24-1.31) | 0.18 |  |
| ARV class experience * No Anchor * Other NRTI+3TC/FTC | 0.67 (0.34-1.32) | 0.25 |  |
| ARV class experience * boosted PI Anchor * TDF only | 0.41 (0.19-0.87) | 0.02 | * |
| ARV class experience * Other Anchor * TDF only | 0.58 (0.25-1.35) | 0.20 |  |
| ARV class experience * No Anchor * TDF only | 0.68 (0.35-1.30) | 0.24 |  |
| ARV class experience * boosted PI Anchor * 3TC/FTC only | 0.44 (0.14-1.34) | 0.15 |  |
| ARV class experience * Other Anchor * 3TC/FTC only | 0.88 (0.27-2.90) | 0.83 |  |
| ARV class experience * No Anchor * 3TC/FTC only | 1.19 (0.35-4.03) | 0.78 |  |
| ARV class experience * boosted PI Anchor * Other NRTI only | 0.55 (0.25-1.23) | 0.14 |  |
| ARV class experience * Other Anchor * Other NRTI only | 0.68 (0.29-1.62) | 0.39 |  |
| ARV class experience * No Anchor * Other NRTI only | 0.81 (0.41-1.62) | 0.55 |  |
| ARV class experience * boosted PI Anchor * No NRTI | 0.54 (0.25-1.14) | 0.11 |  |
| ARV class experience * Other Anchor * No NRTI | 0.66 (0.29-1.52) | 0.33 |  |
| ARV class experience * No Anchor * No NRTI | 0.72 (0.38-1.37) | 0.32 |  |

***S1 File Table C Legend:*** *HR: Hazard Ratio (95% confidence interval in parenthesis). PDC: percentage of days covered: last 30 days for statins and CVMs, last, 90 days for ARVs, VF: Virologic Failure, Boosted PI: Ritonavir boosted Protease Inhibitor, 3TC: Lamivudine, FTC: Emtricitabine. Modern Anchor: Efavirenz, boosted Darunavir, or Integrase Inhibitor. Gray Shade: Baseline covariates (non-time dependent). Native LDL equivalent: LDL or Non-HDL cholesterol -30mg/dL, whichever was higher off lipid-lowering agents. Treatment Interruption: Time period without any ARVs. P-values: * 0.01-0.05, ** 0.001-0.01, *** <0.001.*

## S1 File Table D:

## HR (95% CI) of First Virologic failure *or* Death during Suppressed Viremia Stratified by HAART Era (Combined Endpoint). N=19,324 – 11,599 Events

| Medication Exposure Mode | Bias Correction | | | Statins | Non-Statin Lipid—lowering Agents | Antihyper-tensives | Cardiac Aspirin |  |
| --- | --- | --- | --- | --- | --- | --- | --- | --- |
| PDC  (30/90d)* | None | | 0.60 (0.56-0.64) | | 0.68 (0.62-0.75) | 0.85 (0.82-0.89) | 0.96 (0.89-1.03) | |
|  |  |  | p<0.001 | | p<0.001 | p<0.001 | p=0.26 | |
|  | Multivariate Adjustment | | 0.84 (0.78-0.91) | | 0.94 (0.85-1.03) | 1.04 (0.99-1.09) | 1.20 (1.10-1.30) | |
|  |  |  | p<0.001 | | p=0.17 | p=0.09 | p<0.001 | |
| Current Use (within 7 d) | None | | 0.62 (0.58-0.66) | | 0.69 (0.63-0.75) | 0.90 (0.86-0.94) | 1.01 (0.94-1.09) | |
|  |  |  | p<0.001 | | p<0.001 | p<0.001 | p=0.71 | |
|  | Multivariate Adjustment | | 0.82 (0.76-0.88) | | 0.87 (0.80-0.95) | 1.07 (1.02-1.12) | 1.22 (1.13-1.32) | |
|  |  |  | p<0.001 | | p=0.002 | p=0.006 | p<0.001 | |
|  | IPW | Truncation |  | |  |  |  | |
|  |  | <5% / >95% | 0.77 (0.71-0.84) | | 0.89 (0.80-0.99) | 1.05 (1.00-1.10) | 1.11 (1.01-1.22) | |
|  |  |  | p<0.001 | | p=0.03 | p=0.03 | p=0.03 | |
|  |  | <1% / >99% | 0.83 (0.76-0.91) | | 0.94 (0.84-1.05) | 1.08 (1.03-1.13) | 1.12 (1.01-1.24) | |
|  |  |  | p<0.001 | | p=0.26 | p=0.002 | p=0.03 | |
|  |  | <0.1%/>99.9% | 0.89 (0.80-0.99) | | 0.95 (0.85-1.07) | 1.09 (1.04-1.14) | 1.12 (1.01-1.25) | |
|  |  |  | p=0.03 | | p=0.44 | p<0.001 | p=0.03 | |

***S1 File Table D legend****: All hazard ratios (HR) are followed by 95% confidence intervals in parenthesis.
PDC: percentage of days covered, IPW: Inverse Probability Weighting for treatment and censoring. *PDC interval 30 days for statins/CVMs and 90 days for ARVs. In PDC mode HR is for 100% use.*

## S1 File Table E:

## HR (95% CI) of First Virologic failure Stratified by HAART Era for individual statin compounds and reference drugs. N=19,324 – 10,534 Events

| Current Use  (last 7 d) | Pravastatin  40% | Simvastatin  23% | Fluvastatin  13% | Rosuvastatin  13% | Atorvastatin  9% | Lovastatin  2% | Non-Statin Lipid-Lowering Agents | Antihyper-tensives | Cardiac Aspirin |
| --- | --- | --- | --- | --- | --- | --- | --- | --- | --- |
| No  Adjustment | 0.63 (0.56-0.71) | 0.49 (0.53-0.57) | 0.71 (0.59-0.86) | 0.42 (0.32-0.56) | 0.76 (0.63-0.93) | 0.55 (0.36-0.83) | 0.76 (0.70-0.83) | 0.82 (0.79-0.86) | 0.88 (0.81-0.95) |
|  | p<0.001 | p<0.001 | p<0.001 | p<0.001 | P=0.007 | P=0.004 | p<0.001 | p<0.001 | p=0.002 |
| Multivariate Adjustment | 0.77 (0.68-0.87) | 0.79 (0.68-0.91) | 0.95 (0.78-1.15) | 0.59 (0.44-0.79) | 0.97 (0.79-1.18) | 0.83 (0.54-1.27) | 0.97 (0.89-1.06) | 1.00 (0.96-1.05) | 1.13 (1.04-1.23) |
|  | p<0.001 | p=0.002 | P=0.58 | p<0.001 | p=0.77 | p=0.39 | p=0.65 | p=0.88 | p=0.004 |

***S1 File Table E legend:*** *All hazard ratios (HR) are followed by 95% confidence intervals in parenthesis.*
